# Elevated IL-12, TNF-*α*, and TNF-*α*/IL-10 Ratios in Stored *Plasmodium falciparum*-Infected Whole Blood: Implications for Safe Haemotransfusion

**DOI:** 10.1155/2020/9394585

**Published:** 2020-10-27

**Authors:** Enoch Aninagyei, Patrick Adu, Alexander Egyir-Yawson, Desmond Omane Acheampong

**Affiliations:** ^1^School of Basic and Biomedical Sciences, University of Health and Allied Sciences, PMB 31, Ho, Volta Region, Ghana; ^2^Department of Medical Laboratory Technology, School of Allied Health Sciences, University of Cape Coast, Cape Coast, Ghana; ^3^Department of Biomedical Sciences, School of Allied Health Sciences, University of Cape Coast, Cape Coast, Ghana

## Abstract

Although *Plasmodium falciparum* infections in blood donors have been reported, the impact of parasitaemia on cytokine levels in stored whole blood has not been explored. This study evaluated the effect of *P. falciparum* parasitaemia on circulating cytokines and their relationship with haematological parameters in banked blood. In this case-control study, two groups of donor whole blood were recruited: *P. falciparum*-infected donors (parasitaemia: 515–1877 parasites/*μ*L) and noninfected blood donors (control). At day 0 (baseline), 7, 14, 21, and 35 of banking circulating cytokine levels of tumor necrosis factor alpha (TNF-*α*), interleukin- (IL-) 12, IL-10, and IL-6 levels and haematological parameters were determined. Kruskal-Wallis test determined differences in weekly cytokine levels while Dunn's post hoc test determined exact significant points. At baseline, the mean TNF-*α* (33.81 pg/mL vs. 22.70 pg/mL), IL-12 (28.39 pg/mL vs. 16.15 pg/mL), IL-10 (51.04 pg/mL vs. 18.95 pg/mL), and IL-6 (71.03 pg/mL vs. 30.89 pg/mL) levels were significantly higher in infected donor whole blood. Significant rate of increase was observed in TNF-*α*, IL-12 levels, and TNF-*α*/IL-10 ratios in infected blood, while decreased levels were observed in IL-10. IL-6 peaked at day 21 and fell below baseline level at day 35. Significant changes in TNF-*α*, IL-12, IL-10, IL-6 levels, and TNF-*α*/IL-10 ratios in infected donor blood were observed 7 days after storage. Unlike in noninfected stored whole blood, TNF-*α*, IL-6, IL-12, and TNF-*α*/IL-10 ratio levels in infected stored whole blood related inversely to haematological parameters (white cells, red cells, platelets, and haemoglobin levels) during storage. However, in both groups, significant direct relationship was observed in IL-10 and haematological parameters. In conclusion, banking of *P. falciparum*-infected donor whole blood may lead to infusion of large quantities of inflammatory cytokines with potential adverse immunological response in recipients.

## 1. Introduction

Blood transfusion is mainly used in the clinical management of emergencies involving patients with life-threatening conditions such as severe anaemia, road accidents, and malignancies. Although haemotransfusion is a life-saving venture, it also poses problems of immunological adverse reactions [1]. Studies in Africa have reported various prevalence of asymptomatic *Plasmodium falciparum* parasitaemia in blood donors. For example, whereas a 10% prevalence of *P. falciparum* infection has been reported in Ghana [2], other studies have reported between 30 and 77.4% prevalence elsewhere in Africa [3, 4]. In addition, in Asia, the respective prevalence of *P. falciparum* parasitaemia in Indian, Chinese, and Thai blood donors is 16.9% [5], 2.13% [6], and 0.27% [7]. *P. falciparum* is the most frequent parasite species identified in blood donors; however, few cases of other species have also been identified [8, 9]. These parasites have been demonstrated to survive in whole blood stored at 2-6°C for close to 28 days [10, 11].

In a recent study in Ghana, *P. falciparum* was reported to progressively reduce total leukocytes and neutrophil count in stored blood. Elevations in plasma haemoglobin percentage, haemolysis, and plasma potassium as well as reduction in red cell count were also reported [12]. However, the impact of *P. falciparum* parasitaemia on circulating cytokine profile of the infected whole donor was not explored. Although the potential toxicity and sequelae of transfused red blood cells (RBCs) remain contentious, it has been unequivocally demonstrated that a distinct series of biochemical changes occur during blood storage that gives rise to a litany of chemical and cellular biomolecules [13]. In view of the aforementioned, this study was designed to investigate storage-related changes in circulating tumor necrosis factor alpha (TNF-*α*), interleukin- (IL-) 12, IL-10, and IL-6 in *P. falciparum*-infected donor blood during storage. Whereas TNF-*α*, IL-6, and IL-12 were chosen based on their known proinflammatory activity, IL-10 was chosen because of its demonstrable anti-inflammatory and immunoregulation in *P. falciparum* infections [14, 15].

## 2. Materials and Methods

### 2.1. Study Design

This experimental case-control study was done in sterile donors' whole blood. The donors' whole blood used in this study was collected from the Greater Accra Region of Ghana in collaboration with the National Blood Service, Ghana. Blood donors were recruited from April to December 2018.

### 2.2. Donor Selection Criteria

Eligible donors were selected according to the National Blood Service, Ghana criteria which conforms to the World Health Organization Medical History Guide for Donor Selection protocol [16]. The donors' whole blood included in the study was transfusion transmissible infection- (TTI-) negative blood with baseline (day 0) total leukocyte count between 4.0 and 8.0 × 10^9^/L for both *P. falciparum*-infected (cases; age range: 18–58 years) and non-*P. falciparum*-infected (controls; age range: 21–57 years) donor blood.

### 2.3. Donor Blood Collection

Veins located at the antecubital fossa were made more prominent with the aid of tourniquet. The skin around the selected vein was disinfected with 70% alcohol and allowed to dry. Venipuncture was performed using 16-gauge needle attached to the blood collection bag. As soon as blood flow was established, the tourniquet was removed. About 40 mL of whole blood was collected into the sample pouch attached to the main blood bag (to be used to screen for infectious markers). The blood was then rechanneled into the main blood bag with calculated amount of citrate phosphate dextrose adenine (CPDA-1) anticoagulant. After adequate blood has been collected, the needle was removed, injection site dressed, the needle was severed from the collected tube, blood labelled, and kept on ice packs.

### 2.4. Sample Size Determination

The minimum number of *P. falciparum*-infected blood donors was determined by using the formula: *n* = *z*^2^*p*(1 − *p*)/*d*^2^, where *n* = sample size, *p* = proportion of Greater Accra Region residents reported to be infected with *P. falciparum* by microscopy (*p* = 7.4% [17]), *z* = confidence level at 95% (standard value of 1.96), and *d* = margin of error at 5% (standard value of 0.05). Sample size was calculated as 106.

### 2.5. Choosing Comparative Control Group

To reduce confounding variables to the barest minimum, gender was closely matched. The same number of males and females found to be infected with *P. falciparum* on each donation day were selected for the comparative groups.

## 3. Laboratory Procedures

### 3.1. Screening for Malaria Parasites and Parasitaemia Determination

On the same day of blood collection, donor blood was screened for *P. falciparum* infection using PfHRP-2/pLDH SD Bioline rapid diagnostic test kit (Gyeonggi-do, Republic of Korea). Malaria parasitaemia was determined in PfHRP2/pLDH positive donor whole blood that satisfied the inclusion criteria. Malaria parasitaemia was determined with 3% Giemsa staining as described elsewhere, and parasitaemia was estimated by dividing the number of asexual parasites per at least 200 leukocytes and multiplied by estimated WBC of 8000 cells/*μ*L of blood [18].

### 3.2. Donor Screening for Transfusion-Transmitted Infections

Each donor whole blood was screened for other transfusion-transmitted infections (hepatitis B virus, hepatitis C virus, HIV I&II, and *Treponema pallidum*) using fourth generation ELISA (Abnova, Taiwan) as previously described [12].

### 3.3. Experimental Design

The samples were collected for cytokine measurement on the day of donor phlebotomy (day 0; baseline) and on day 7, day 14, day 21, day 28, and day 35 of whole storage at an average temperature of 4-8°C. On each analysis day, 5 mL of well-mixed CPDA-1-anticoagulated whole blood was aseptically aspirated using 22G Vacuette® multiple-use drawing needle (Greiner Bio-One, Austria) into a plain vacutainer tube. The whole blood was spun at 2000 rpm for 10 minutes. About 1.5 mL of plasma was aspirated into cryovial tube and stored frozen at -30°C using TSX series, Thermo Scientific^TM^ Freezer (USA) until ready for cytokine assay.

### 3.4. Haematological Profiling

Absolute white cell count, red blood cell count, platelets, and haemoglobin levels were done on automated haematology analyzer (Urit 5160, China). The analyzer works on the principle of laser beam multidimensional cell classification, flow cytometry for white cell count, and differentiation and haemoglobin concentration were measured by cyanide-free colorimetric method.

### 3.5. Sandwich ELISA for TNF-*α*, IL-10, IL-12, and IL-6

Quantitative ELISA for human TNF-*α* (ab181421), human IL-10 (ab100549), human IL-6 (ab46027), and human IL-12 (ab46035) were done using Abcam sandwich ELISA kit (Abcam Trading Shanghai Company Ltd., Pudong, Shanghai, China) in accordance with manufacturer's protocol. The detection limits for human TNF-*α*, human IL-10, human IL-12, and human IL-6 were 1.4 pg/mL, <1 pg/mL, 0.75 pg/mL, and <2 pg/mL, respectively.

### 3.6. Data Processing and Statistical Analysis

Baseline and weekly data were entered into Microsoft Excel 2016. Statistical analysis was done by SPSS Version 24 (Chicago, IL, USA). Differences between baseline and weekly cytokine levels were determined by Kruskal-Wallis nonparametric test while Dunn's post hoc test determined exact significant points. Analysis of differences in cytokine levels in *P. falciparum* infected and noninfected donor blood for each week was determined by Mann-Whitney *U* test. *P* value of <0.05 was considered statistically significant.

## 4. Results

### 4.1. Characteristics of the Infected and Noninfected Blood Donors

A total of 230 donor samples were collected; 115 were infected with *P. falciparum* while another 115 randomly selected were noninfected donor blood. Gender was strictly matched (for both donor groups, 74.7% were males and the rest were females). There were no significant differences between the mean age of the *P. falciparum* infected and noninfected donors (*P* = 0.118). In both donor groups, more than half were of blood group O positive (infected donors, 68.7%; noninfected donors, 57.4%). The rest were either blood type A or B positive. While blood pressure values were significantly higher in infected donors, the reverse was observed for platelet counts. Again, haemoglobin, red blood cell, and white blood cell levels were insignificantly lower in infected donors (Table 1).

### 4.2. Changes in Cytokine Levels in Donor Whole Blood Stored for 35 Days

**Figure 1:**
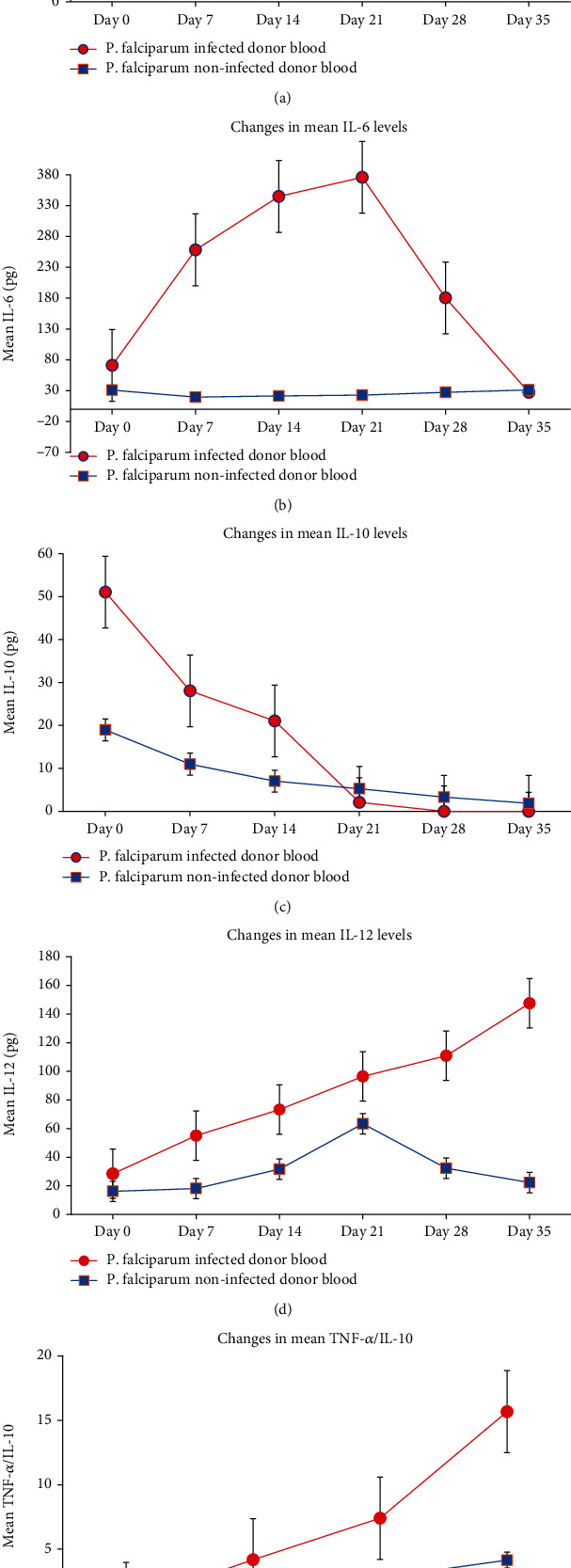
Changes in cytokine levels in donor whole blood stored for 35 days: (a) changes in TNF-*α* levels, (b) changes in IL-6 levels, (c) changes in IL-10 levels, (d) changes in IL-12 levels, and (e) changes in TNF-*α*/IL-10 levels. *t*-test determined *P* values to be <0.05 in all cytokine levels for each timed point after storage between infected and noninfected donor blood.

Figure 1 represents the changes in measured cytokine levels in donor whole blood stored for 35 days. Baseline mean levels of all measured cytokines were significantly higher in malaria-infected donor blood than levels in noninfected donor whole blood (Figures 1(a)–1(e)). Even though mean tumor necrosis factor alpha (TNF-*α*) in noninfected donor blood increased from 22.70 pg/mL at day 0 to 185.05 pg/mL at day 35 (*P* < 0.05; Figure 1(a)), higher rate of progressive increments was observed in infected donor whole blood during storage. Similarly, in the malaria-infected donor blood, TNF-*α* increased progressively by 192.0% at day 7 to 433.3% at day 35 (*P* < 0.05). However, at days 28 and 35, IL-10 was not detected in infected donor blood. Additionally, the mean IL-6 sharply increased in malaria-infected donor blood, peaking at day 21 and then sharply decreased to levels in nonmalaria-infected donor blood (Figure 1(b)). However, in malaria-noninfected donor blood, IL-6 levels decreased below baseline levels at day 7 (36.7% decrease) and stayed below baseline values until day 35. IL-10 levels dropped from baseline values in both donor groups, and the levels stayed significantly higher in malaria-infected donor blood until day 21 when IL-10 levels dropped below the levels in nonmalaria-infected blood (Figure 1(c)). Furthermore, the mean IL-12 levels in noninfected donor blood increased progressively at day 0 (16.15 pg/mL), peaked at day 21 (63.32 pg/mL), and decreased to 32.3 pg/mL at day 28 and further to 22.29 pg/mL at day 35 (*P* < 0.05) (Figure 1(d)). In the infected group, IL-12 almost doubled at day 7 and increased progressively by 94.0% at day 7 to 419.47% at day 35 (*P* < 0.05). Moreover, TNF-*α*/IL-10 ratio increased exponentially in malaria-infected donor blood, while in the noninfected donor blood, marginal elevations were recorded (Figure 1(e)). TNF-*α*/IL-10 ratios after day 21 were incalculable due to undetected levels of IL-10 in day 28 and day 35.

### 4.3. Changes in Haematological Parameters during Storage

**Figure 2:**
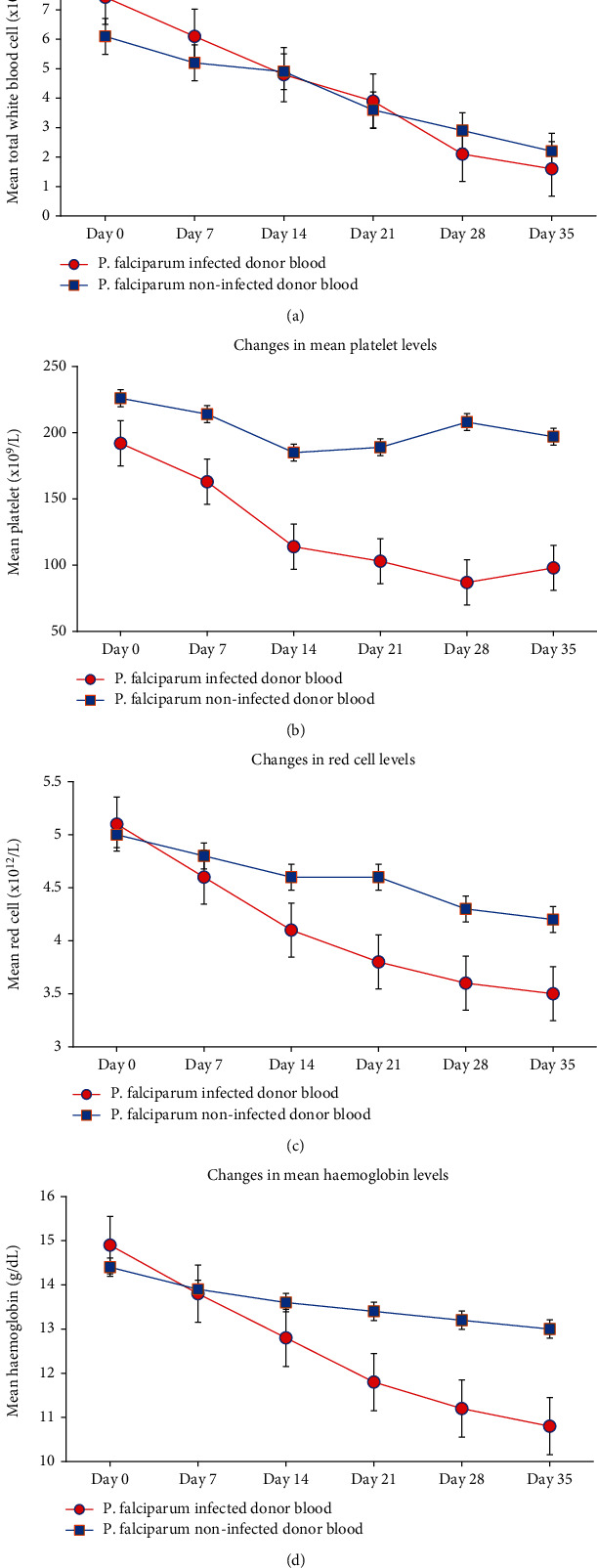
Changes in haematological parameter levels in donor whole blood stored for 35 days: (a) changes in total white blood cell levels, (b) changes in platelet levels, (c) changes in red blood cell levels, and (d) changes in haemoglobin levels.

Figure 2 shows the changes in total white blood cells, platelets, red blood cells, and haemoglobin levels. It was observed that total white cell levels decreased in both infected stored whole blood and in control set-up. However, mean total white cell levels in infected donor blood were significantly lower than control samples at baseline, day 28, and day 35 (Figure 2(a)). Also, in infected donor blood, platelet levels decreased gradually from day 0 to day 35 compared to control levels (Figure 2(b)). Even though red blood cells and haemoglobin levels decreased in both infected donor blood and control samples, significant higher rate of changes was observed in infected donor blood than noninfected donor blood (Figures 2(c) and 2(d)).

### 4.4. Relationship between Circulating Cytokines and Haematological Parameters

Inverse relationship was observed in TNF-*α*, IL-6 (up to day 21), IL-12, and TNF-*α*/IL-10 ratio (Figure 1) as well as in the haematological parameters (Figure 2). Whereas nonsignificant relationship was observed in noninfected control samples, significant correlations were observed in infected donor blood during storage. However, in both study groups, significant direct relationship was observed in IL-10 and haematological parameters.

### 4.5. Conceptualization of Cytokine Levels in an Average of 500 mL of Stored Infected Whole Blood

The amounts of cytokines that accumulated in stored whole blood during the 35-day storage period were quantified (Table 2). Whereas transfusing a 500 mL malaria-infected donor whole blood stored for 35 days could result in inadvertently infusing 90.2 ng or 73.7 ng of TNF-*α* and IL-12, respectively, transfusing an equivalent volume of nonmalaria-infected donor whole blood stored for 35 days may lead to infusing 32.4 ng or 11.1 ng of TNF-*α* and IL-12, respectively. Furthermore, whereas transfusing a 500 mL malaria-infected donor whole blood stored for 21 days may result in infusing 190 ng of IL-6, transfusing an equivalent volume of nonmalaria-infected blood will result in infusing only 11.4 ng of IL-6.

## 5. Discussion

The core mandate of blood banks is to provide safe and timely blood and blood component(s) to recipients to improve their physiological status and disease treatment outcomes [19]. However, prolonged storage of donor blood allows for untoward biochemical changes in donor blood [12]. In this case-control study, the storage changes of interleukin- (IL-) 6, IL-10, IL-12, and tumor necrosis factor alpha (TNF-*α*) in donor blood infected with *Plasmodium falciparum* were compared with noninfected controls. In both cases and controls, samples were negative for hepatitis B virus, hepatitis C virus, syphilis, and HIV I&II. This was to ensure that the levels of IL-6, IL-10, IL-12, and TNF-*α* were not compounded by that of the screened transfusion-transmitted infections. Therefore, the baseline levels of analyzed cytokines were solely as a result of *P. falciparum* infections. In this study, it was confirmed that the baseline levels of inflammatory cytokines IL-6, IL-10, IL-12, and TNF-*α* in malaria-infected donor blood (parasitaemia, 515-1877 parasites/*μ*L of blood) were significantly higher compared to noninfected controls. These findings were consistent with previous studies on asymptomatic *P falciparum* infections [20–22]. Noteworthily, our study demonstrates significantly increased production of these cytokines in *P. falciparum*-infected blood and thus argues against transfusion of such donor blood. In a previous study, without *P. falciparum* contaminations, leukocytes were established to release cytokines during whole blood and concentrated red cell storage for 35 days. However, higher levels were observed in whole blood compared to red cell concentrates [23, 24]. Exaggerated increases were observed in stored infected whole blood.

Previous studies have found the average age of blood transfused to patients to range from 16 to 21 days [25]. Our study estimates that transfusing a 500 mL malaria-infected stored whole blood for 21 days could result in infusing 190 ng, 71.6 ng, or 48.2 ng of IL-6, TNF-*α*, and IL-12, respectively, to the recipient. When our study outcome is considered in the light of the reported general average of 2 to 5 donor blood transfused per patient [25, 26], our study is suggestive that substantial quantities of these inflammatory cytokines are likely to be introduced into the recipients of malaria-infected donor blood with obvious negative clinical consequences. Taken together with the fact that a significant proportion of transfusion recipients in sub-Saharan Africa may be infected with severe malaria, transfusion of such a stored whole blood may precipitate a cytokine storm which may potentially worsen patient treatment outcomes. TNF-*α* is known to induce fever *in vivo*, either directly through stimulation of prostaglandin E_2_ (PGE_2_) synthesis or indirectly by inducing release of IL-1. TNF-*α* also stimulates a proinflammatory cytokine, IL-6, synthesis in several immune cell types [27]. The combined effect of IL-6 and TNF-*α* in the induction of fever could induce and exacerbate preexisting fever through a cascade of cytokines with overlapping properties. Again, TNF-*α* is also known to induce hemorrhagic necrosis *in vivo* [28]. For these reasons, malaria-infected donor whole blood stored for more than 7 days may not be suitable for haemotransfusion as it may induce either immediate or delayed posttransfusion reaction. IL-6 levels were observed to peak at day 21 following gradual accumulation during storage of *P. falciparum*-infected blood. Functionally, IL-6 has been reported to promote inflammation and pyrexia. Previous studies have implicated IL-6 as a biomarker for propagation of chronic inflammation by promoting mononuclear cell accumulation [29]. It has also been known to play a prominent role in inducing fever in response to both endogenous and exogenous pyrogens [30]. Noteworthily, the levels of most of the cytokines declined after 21 days, suggesting either the loss of signal for the cytokine production or cell(s) that produced these cytokines were lost post 21 days. Although we did not explore the causality of the decline in inflammatory cytokines in the present study, future studies will explore the mechanisms as well as clinical implications in transfusion recipients. In this study, baseline IL-12 levels were found to be higher in infected blood donors than noninfected donors. This observation was not surprising because IL-12 is one of the few proinflammatory cytokines that are released in response to early infections [31]. In addition, as observed in this study, IL-12 and IL-10 were negatively correlated as confirmed by an earlier study [32]. During storage, IL-12 levels increased progressively from baseline to day 35. Again, it was observed that, when a single *P. falciparum*-infected whole blood (approximately 500 mL) is transfused on the same day of blood collection, 14195 pg (14.2 ng) of IL-12 will theoretically be infused into the blood recipient. IL-12 levels increased significantly to 36850 pg (36.9 ng) when stored for 35 days. When the results reported herein are considered in the light of previous studies, one could argue for a potential beneficial effect of high levels of IL-12 in stored *P. falciparum*-infected whole blood in blood recipients. For example, both in animal models and in human experimental studies, IL-12 has been found to mediate cytotoxic effects of NK cells through IFN-*γ* expression by promoting mitosis and antiangiogenic effects of T cells [33]. Again, IL-12 is an essential factor in resistance to bacterial and intracellular parasitic infection. IL-12 has shown significant progress in the treatment of malignant diseases in view of its reported antitumor activity [34]. Furthermore, through generation of IFN-*γ*, excitation of NK cells, and lymphokine-activated killer cell, IL-12 has been demonstrated to enhance clearance of HCV from infected hosts [35]. Other studies have also shown potential roles of IL-12 in hepatitis B virus (HBV) infection [36] as well as influenza virus infection [33]. In spite of these previous benefits of high levels of IL-12, the fact that *P. falciparum* causes red cell haemolysis argues against any such potential benefits of transfusing *P. falciparum*-infected donor blood. Exponential increase in TNF-*α*/IL-10 ratio in infected donors was observed. Accumulation of TNF-*α* and exponential increase in TNF-*α*/IL-10 ratio was a result of inhibition of IL-10 in the infected donor blood. This is because overproduction of TNF-*α* and suppression of IL-10 have been found to inhibit erythropoietin activity [37]. High TNF-*α*/IL-10 ratio has also been associated with anaemia due to malaria in an earlier study [38]. Based on these findings, banking *P. falciparum*-infected blood could exacerbate preexisting anaemia in blood recipients, through inhibition of erythropoietin activity by high TNF-*α* levels and low IL-10 levels.

A key limitation of the present study was the fact that it was undertaken in stored whole blood. We are therefore not able to provide any data as to whether component separation at the point of blood donation could have impacted cytokine levels in all blood components in the same way. In spite of this limitation, our study demonstrates that transfusing of *P. falciparum*-infected whole blood stored for over 7 days could be inimical to the recipient. Accumulation of proinflammatory cytokines such as TNF-*α*, IL-6, and IL-12 could cause nonhaemolytic febrile reactions in the recipients [39]. Cytokines induced by posttransfusion febrile reactions may include fever, chills, headache, myalgia, and general malaise. Hypotension, vomiting, and respiratory distress may also occur occasionally. These reactions may occur during or several hours after transfusion, and the severity of the reaction may be dependent upon leucocyte load of the blood and the rate and frequency of transfusion [40, 41].

Severe storage lesions characterized by reduction in total white cells, red blood cells, platelets, and haemoglobin were observed in infected donor blood. These haematological parameters were found to be related inversely to TNF-*α*, IL-12, and IL-6. Previously, it was established that reduction in haematological parameters during blood storage could be as a result of histamine, lipids, and cytokines released by leucocytes in the storage medium [13]. In this study, TNF-*α*, IL-12, and IL-6 have been shown to be responsible for the reduction of the haematological parameters during blood banking and significantly in infected donor blood. Furthermore, the accumulation of biomolecules such as cytokines may induce febrile transfusion reactions with its attendant increased membrane damage and suppressing the immune system [42].

## 6. Conclusion

In malaria-endemic areas, screening for malaria parasites at the point of donor recruitment and subsequent deferral of asymptomatic malaria-infected donors should be mandatory as transfusion of such stored whole blood may lead to inadvertent infusion of large quantities of TNF-*α*, IL-6, and IL-12 cytokines with potential adverse immunological events. Also, accumulation of these cytokines contributed to reductions in vital haematological cells which are supposed to be therapeutic to the blood recipient. Assessment of changes in cytokine levels in stored *P. falciparum*-infected blood in separated components is recommended for future study. Furthermore, if stored whole blood is essential, cytokine levels in buffy coat free infected whole blood should also be studied to determine the changes in cytokine levels.

## Figures and Tables

**Table 1:** Characteristics of the blood donors.

Parameters	Infected donors (*n* = 115)	Noninfected donors (*n* = 115)	*P* value
Age			
Mean age in completed years	31 ± 9.2	33 ± 10.1	0.118^d^
Gender			1^b^
Male	86 (74.8%)	86 (74.8%)	
Female	29 (25.2%)	29 (25.2%)	
Red cell phenotypes			0.153^c^
O positive	79 (68.7%)	66 (57.4%)	
A positive	25 (21.7%)	30 (26.1%)	
B positive	11 (9.6%)	19 (16.5%)	
^a^Systolic pressure (mmHg)	125.7 ± 6.3	123.5 ± 6.9	0.012^d^
^a^Diastolic pressure (mmHg)	78.4 ± 4.8	76.8 ± 2.7	0.002^d^
^a^Haemoglobin (g/dL)	15.1 ± 1.9	14.3 ± 2.3	0.073^d^
^a^Total white cell count (cells/*μ*L)	7822 ± 1316	6315 ± 841	0.115^d^
^a^Red cell counts (cells/*μ*L)	5.13 ± 1.11 × 10^6^	5.08 ± 1.28 × 10^6^	0.206^d^
^a^Platelet counts (cells/*μ*L)	193 ± 79 × 10^3^	235 ± 118 × 10^3^	<0.05^d^

^a^Baseline values presented as mean ± standard deviation. ^b^Chi-square statistic with Yates correction is 0.023, *P* = 0.878. ^c^Chi-square statistic is 3.75. ^d^*P* value determined by the Student *t*-test.

**Table 2:** Amounts of cytokines released into an average of 500 mL of stored whole blood.

	Mean levels per 500 mL infected donor blood					
Cytokines	Day 0	Day 7	Day 14	Day 21	Day 28	Day 35
Malaria-infected donor blood						
IL-10 (ng)	25.5	14.0^∗^	10.5^∗^	1.1^∗^	0.0	0.0
IL-6 (ng)	35.5	130^∗^	170.0^∗^	190.0^∗^	100.0^∗^	13.5^∗^
TNF-*α* (ng)	16.9	49.3^∗^	65.6^∗^	71.6^∗^	75.1^∗^	90.2^∗^
IL-12 (ng)	14.2	27.5^∗^	36.6^∗^	48.2^∗^	55.4^∗^	73.7^∗^
Malaria-noninfected donor blood (control)						
IL-10 (ng)	9.5	5.5^∗^	3.5^∗^	2.6^∗^	1.7^∗^	0.9^∗^
IL-6 (ng)	15.4	9.7^∗^	10.8^∗^	11.4^∗^	13.7^∗^	15.6^∗^
TNF-*α* (ng)	11.4	13.4^∗^	15.0^∗^	21.3^∗^	29.4^∗^	32.4^∗^
IL-12 (ng)	8.1	9.18^∗^	15.8^∗^	31.7^∗^	16.2^∗^	11.1^∗^

Abbreviations: ng: nanogram (10^−9^ gram); ^∗^Dunn's post hoc test indicated significant levels from mean day 0 levels.

## Data Availability

The datasets generated and/or analyzed during the current study are available in Harvard Dataverse repository: 10.7910/DVN/CPLADC.
